# Effect of Retrogression with Different Cooling Ways on the Microstructure and Properties of T’/η’ Strengthened Al-Zn-Mg-Cu Alloys

**DOI:** 10.3390/ma17081746

**Published:** 2024-04-11

**Authors:** Jianlei Zhang, Guwei Shen, Bingzhuo Han, Dayong Li, Zhenyu Xu, Zhenlong Chao, Guoqin Chen, Longtao Jiang

**Affiliations:** 1 School of Materials Science and Engineering, Harbin University of Science and Technology, Harbin 150040, China; 2 School of Materials Science and Engineering, Harbin Institute of Technology, Harbin 150001, China; 3 State Key Laboratory of Advanced Welding and Joining, Harbin Institute of Technology, Harbin 150001, China

**Keywords:** Al-Zn-Mg-Cu alloys, T’/η’ phase, retrogression and re-aging, retrogression cooling ways, mechanical property, corrosion resistance

## Abstract

Retrogression and re-aging (RRA) treatment has been proven to effectively overcome the trade-off between strength and corrosion resistance. Current research focuses on the heating rate, temperature, and holding time of retrogression treatment while ignoring the retrogression cooling ways. In this paper, the effects of RRA treatment with different retrogression cooling ways on the microstructure and properties of newly developed T’/η’ strengthened Al-Zn-Mg-Cu alloys were investigated by performing tests on mechanical properties, intergranular corrosion (IGC) resistance, and electrochemical corrosion behavior. The results show that the mechanical properties of samples subject to RRA treatment with water-quenching retrogression (ultimate tensile strength, yield strength, and elongation of 419.2 MPa, 370.2 MPa, and 15.9, respectively) are better than those of air-cooled and furnace-cooled samples. The corrosion resistance of water-quenching (IGC depth of 162.2 μm, corrosion current density of 0.833 × 10^−5^ A/cm^2^) and furnace-cooled samples (IGC depth of 123.7 μm, corrosion current density of 0.712 × 10^−5^ A/cm^2^) is better than that of air-cooled samples. Microstructure characterization reveals that the effect of the retrogression cooling rate on mechanical properties is related to the size of T’/η’ precipitates with grains as well as the proportion of T’ and η’, while the difference in corrosion resistance depends on the continuity of grain boundary precipitates (GBPs). With mechanical properties, corrosion resistance, and time cost taken into consideration, it is appropriate to select water quenching for retrogression. These findings offer valuable insights for further design to achieve superior performance in various applications.

## 1. Introduction

Arising from the emergence of environmental challenges, such as increasing energy consumption and global warming, the development of high-performance lightweight materials is particularly urgent [1,2,3]. Al-Zn-Mg-Cu alloys have been extensively used as lightweight materials in the automotive and aerospace industries due to their exceptional specific strength, superior processability, and recyclability [4,5]. The high strength arises from the large number of dispersed nano-sized precipitates within grains [6,7]. Despite the advantages, the significant potential difference between grain boundary precipitates (GBPs) and the aluminum matrix, as well as the continuously distributed GBPs, results in poor resistance to intergranular corrosion (IGC) [8], limiting their further application potential. The properties of heat-treatable aluminum alloys are governed by various microstructure features, including characteristics of precipitates within grains (type, morphology, size, and number density), precipitates-free zones (PFZs), and GBPs [9]. It is crucial to carefully control the precipitation during aging to achieve optimal comprehensive properties in Al-Zn-Mg-Cu alloys. Micro-alloying serves as an effective approach for modifying the microstructure and improving the properties of Al-Zn-Mg-Cu alloys [10]. Previous studies have demonstrated that the trace addition of Sc [11,12], Er [13,14], and Ag [15,16] has positive effects on the strength and corrosion resistance of Al-Zn-Mg-Cu alloys. However, the high price of these alloying elements hinders the widespread industrial application. In recent years, T’-Mg_32_(Al, Zn, Cu)_49_/η’-MgZn_2_ strengthened Al-Zn-Mg-Cu alloys have received great attention. The T’ phase is recognized as the main strengthening precipitate in Al-Zn-Mg-Cu alloys featuring lower Zn/Mg ratios [17]. Studies [18,19,20,21] on T’ phase-strengthened Al-Mg-Zn-Cu alloys have revealed the remarkable potential for age-hardening responses. Moreover, the reduced potential difference between T’ phases at grain boundaries and the Al matrix enhances corrosion resistance compared to η’ phase-strengthened alloys [22,23,24]. The proportion of T’ and η’ phases can be tailored by manipulating the Zn/Mg ratio [25] and the aging treatment process [26], yielding Al-Zn-Mg-Cu alloys with superior overall performance. However, a challenge arises from the continuous distribution of GBPs in the T6 state, which is susceptible to serving as the corrosion channel.

Heat treatment also has a great effect on the microstructure and properties of heat-treatable Al-Zn-Mg-Cu alloys. The discontinuous distribution of GBPs under the over-aging state is beneficial for improving the IGC resistance of alloys but at the cost of losing mechanical properties [27]. Thermomechanical treatment (TMT) is an effective method to improve the strength. However, there is no consensus on the effect of TMT on the corrosion resistance of Al-Zn-Mg-Cu alloys. The relatively high temperature of artificial aging accelerates the abasement of dislocations, weakening the strengthening effect [28]. Several studies have revealed that non-isothermal aging with suitable process routes and parameters can synergistically improve strength and corrosion resistance [29]. Nevertheless, the determination of suitable process routes and parameters is complex and difficult to take into control. Retrogression and re-aging (RRA) treatment has been proven effective in improving corrosion resistance without sacrificing mechanical properties [30,31]. RRA treatment encompasses three stages of aging treatment, with the first and third stages being equivalent to peak aging. The high-temperature regression treatment is the key to the entire process. Existing studies concentrated on examining the influence of heating rate [32,33], temperature [34,35], and duration [36,37] during the retrogression treatment, while neglecting the effect of retrogression cooling ways. Moreover, the current research mentioned above is all based on η’-strengthened Al-Zn-Mg-Cu alloys. Appropriate RRA treatment for T’/η’ phase-strengthened Al-Zn-Mg-Cu alloys remains to be developed.

The aim of this study is to investigate the effect of retrogression cooling ways during RRA treatment on T’/η’ phase-strengthened Al-Zn-Mg-Cu alloys. The size, morphology, and distribution of precipitates within grains and along grain boundaries were observed and characterized in detail. The mechanical and corrosion properties after RRA treatments with different retrogression cooling ways were compared through tensile tests, IGC depth, and electrochemical tests. Additionally, relationships between mechanical properties, IGC behavior, and microstructure evolution after RRA treatments with varying retrogression cooling rates have been established. These findings offer valuable insights into the optimization of RRA treatments for T’/η’ phase-strengthened Al-Zn-Mg-Cu alloys with an exceptional balance of strength and IGC resistance.

## 2. Materials and Methods

### 2.1. Materials

**Figure 1:**
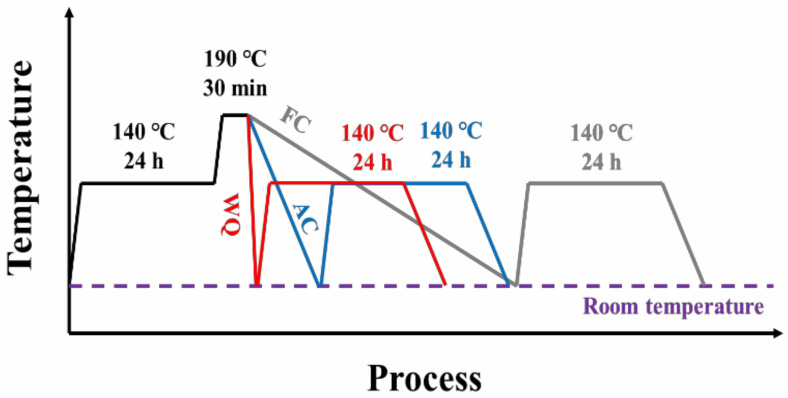
Schematic diagram of RRA treatments with different cooling ways.

The experimental Al-Zn-Mg-Cu alloy was supplied by Fujian Xiangxin Co., Ltd. (Fuzhou, China), which underwent casting at 710 °C, homogenization (475 °C, 24 h), and scalping to 150 mm in diameter. The nominal chemical composition range is presented in Table 1. The samples were subjected to solution treatment at 475 °C for 1 h in an air furnace, followed by immediate water quenching and subsequent RRA treatments. After retrogression treatment, samples were cooled in three different ways: water quenching (WQ), air cooling (AC), and furnace cooling (FC). Details of RRA treatments are shown in Figure 1 and Table 2. The cooling rates of WQ, AC, and FC are over 600 °C/s, 10~15 °C/min, and 1~2 °C/min, respectively.

### 2.2. Mechanical Tests

The tensile test specimens possess a length of 75 mm and a width of 10 mm. Tensile tests were conducted using a universal testing machine (Instron 5569, Norwood, MA, USA) at a constant tensile rate of 1 mm/min. Each reported testing result is representative of the average of at least three individual samples.

### 2.3. IGC Test

The IGC tests were performed according to the GB/T 7998-2005 standard [38]. The samples were ground using 2000# fine sandpaper followed by ultrasonication in an ethanol solution. Subsequently, a washing step was carried out in 10 wt.% NaOH, followed by rinsing in 30 vol% HNO_3_. Finally, the IGC tests were conducted in the corrosion solution (containing 57 g NaCl, 10 mL H_2_O_2_ (ρ = 1.1 g/mL), and 1 L H_2_O) at 35 ± 2 °C for 6 h. Three parallel samples were used for IGC tests.

### 2.4. Electrochemical Test

A three-electrode configuration was used for electrochemical tests. The reference electrode, counter electrode, and working electrode are a saturated calomel electrode (SCE, 232-01, Rex Electric Chemical Ltd., Miami, FL, USA), a platinum tablet (SED-75, TOYO Co., Ltd., Tokyo, Japan), and samples with an exposed area of 100 mm^2^, respectively. Before the test, samples were ground and polished. To ensure a stable state, the open circuit potential (OCP) was recorded for 900 s before the polarization tests. Polarization curves were obtained in a 3.5 wt.% NaCl aqueous solution, ranging from −1.8 V to 0.2 V, with a consistent scan rate of 5 mV/s. Each reported value represents the average of at least three individual measurements.

### 2.5. Microstructure Observation

The maximum corrosion depth of samples after IGC tests were observed and determined from the cross-section using the scanning electron microscope (SEM, Quanta 200FEG, Hillsboro, OR, USA).

A transmission electron microscope (TEM, FEI Talos F200X, Valley City, ND, USA) was employed to capture the bright-filed (BF) images and high-resolution transmission electron microscope (HRTEM) images, especially the characteristics of precipitates within grains and along grain boundaries. Samples were mechanically ground to achieve a thickness of approximately 50 μm, followed by double jet electro-polished in the electrolyte solution (25 vol% nitric acid and 75 vol% methanol) at a temperature of −35 °C and a current of 60 mA. According to the acquired BF images, Nano Measure 1.2 software was used to assess the dimension and quantity of precipitates, with over 300 precipitates analyzed for each condition.

## 3. Results

### 3.1. Microstructure

**Figure 2:**
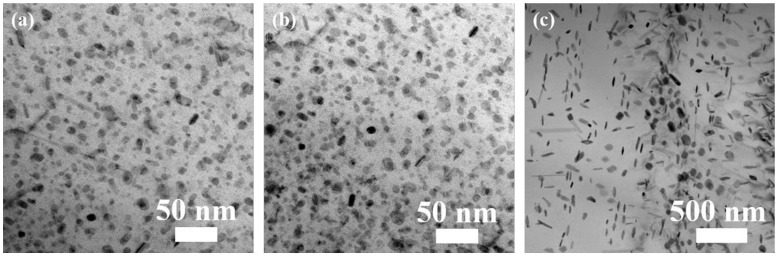
BF images of precipitates within the grain along [110]_Al_ in samples after RRA treatments with different retrogression cooling ways: (**a**) WQ, (**b**) AC, and (**c**) FC.

Figure 2 shows the BF TEM images of precipitates within the grain in samples subjected to RRA treatments with varying retrogression cooling ways. Both plate-like and round-shaped precipitates can be clearly observed. Detailed size information is listed in Table 3. As the retrogression cooling rate decreases from WQ to AC, the aspect ratio of plate-like precipitates and the diameter of round-shape precipitates increase to 3.0 and 9.2 nm, respectively. When the retrogression cooling rate is further reduced to FC, the diameter and thickness of plate-like precipitates increase significantly and the aspect ratio also increases to 4.0. Meanwhile, the diameter of round-shape precipitates increases to 61.9 nm. Moreover, it is observed that the proportion of plate-like and round-shaped precipitates is similar after WQ and AC treatments, while the proportion of plate-like precipitates significantly increases after FC treatment.

**Figure 3:**
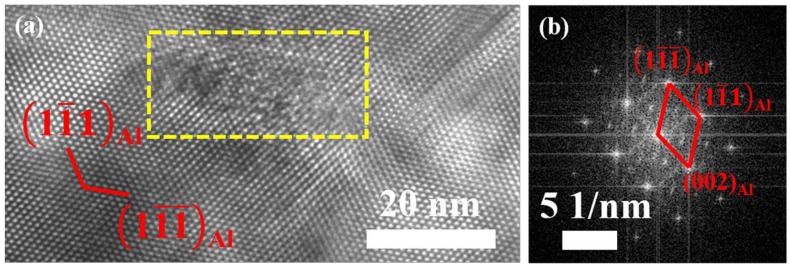

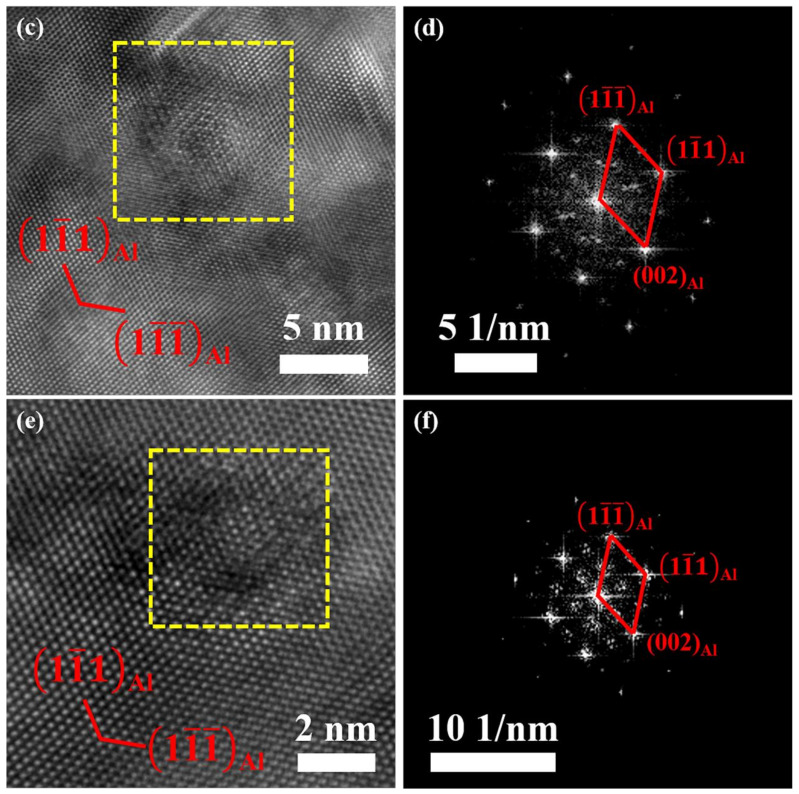
HRTEM images (**a**,**c**,**e**) and corresponding FFT patterns (**b**,**d**,**f**) of precipitates within the grain along [110]_Al_, in the sample subjected to WQ treatment.

Further investigations were conducted to identify plate-like and round-shaped phases. Figure 3a,b present the HRTEM image of the plate-like phase observed in the [110]_Al_ direction, along with the corresponding fast Fourier transform (FFT) pattern. The plate-like phase resides on the habit plane of (111)_Al_ and its diffraction spots are located at 1/3 and 2/3 [220]_Al_, demonstrating the same characteristic of the η’ phase as reported in previous literature [39,40]. Moreover, the orientation relationship between the Al matrix and η’ phase can be identified as [110]_Al_//[$10\overset{-}{1}0$]_η’_. Additionally, some of the round-shape precipitates also exhibit the characteristics of the η’ phase, as shown in Figure 3c,d. As shown in Figure 3e,f, the morphology of the sphere phase in the HRTEM image and the corresponding FFT pattern align well with the characteristics of the T’ phase [41,42,43]. Previous studies have confirmed that the η’ phase exhibits a round shape (face-on) and plate-like (edge-on) [44,45,46] when viewed from [110]_Al_ axes. The projection of the T’ phase maintains a nearly round shape regardless of the zone axis being viewed due to its globular morphology [47,48].

**Figure 4:**
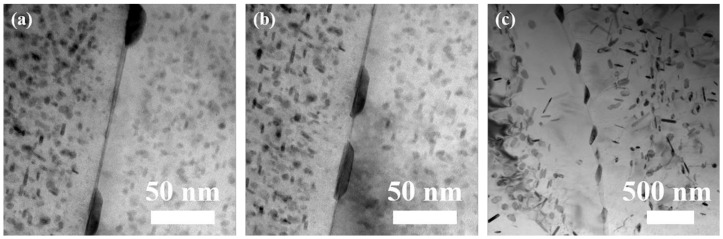
BF images of precipitates along the grain boundary along [110]_Al_ in samples after RRA treatments with different retrogression cooling ways: (**a**) WQ, (**b**) AC, and (**c**) FC.

Figure 4 presents BF TEM images of precipitates along the grain boundary in samples after RRA treatments with different retrogression cooling ways. It is evident that GBPs are distributed discontinuously, effectively hindering the propagation of corrosion cracks. The width between two adjacent GBPs in samples subjected to WQ, AC, and FC treatments are 114.3 nm, 50.0 nm, and 296.3 nm, respectively.

### 3.2. Mechanical Properties

**Figure 5:**
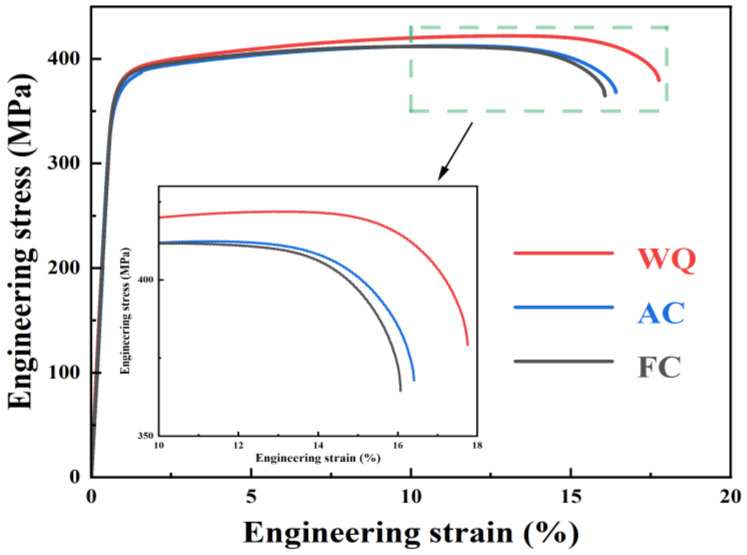
Engineering stress–strain curves of samples after RRA treatments with different retrogression cooling ways.

Figure 5 and Table 4 show the engineering stress–strain curves and tensile property results of specimens subjected to varying RRA treatments. It can be seen that the strength and ductility of samples subjected to WQ treatment are the highest. The ultimate tensile strength (UTS), yield strength (YS), and elongation (EL) are 419.2 MPa, 370.2 MPa, and 15.9, respectively. As the retrogression cooling rate decreases from WQ to AC, the UTS, YS, and EL decrease to 408.3 MPa, 356.2 MPa, and 15.0, respectively. When the retrogression cooling rate is further decreased to FC, the tensile properties remain almost unchanged.

### 3.3. IGC and Electrochemical Tests

**Figure 6:**
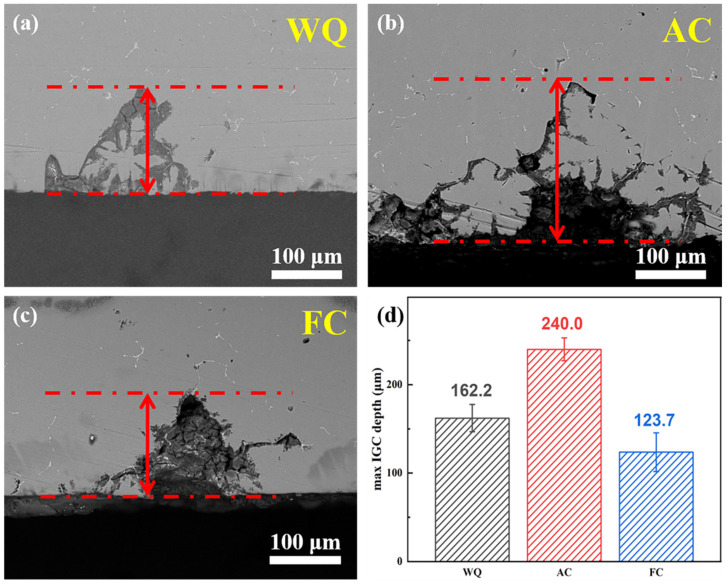
Cross-section SEM images of corrosion samples subjected to RRA treatments with different retrogression cooling ways: (**a**) WQ; (**b**) AC; and (**c**) FC. (**d**) Statistical findings of mean maximum IGC depth.

Figure 6 illustrates the morphology of corrosion samples after various RRA treatments and the corresponding statistical result of the mean maximum IGC depth. The average maximum corrosion depth of samples after WQ, AC, and FC treatments are 162.2 μm, 240.0 μm, and 123.7 μm, respectively. With the decrease in retrogression cooling rate, the corrosion resistance becomes worse and then is improved. Samples subjected to AC treatment exhibit the poorest corrosion resistance.

**Figure 7:**
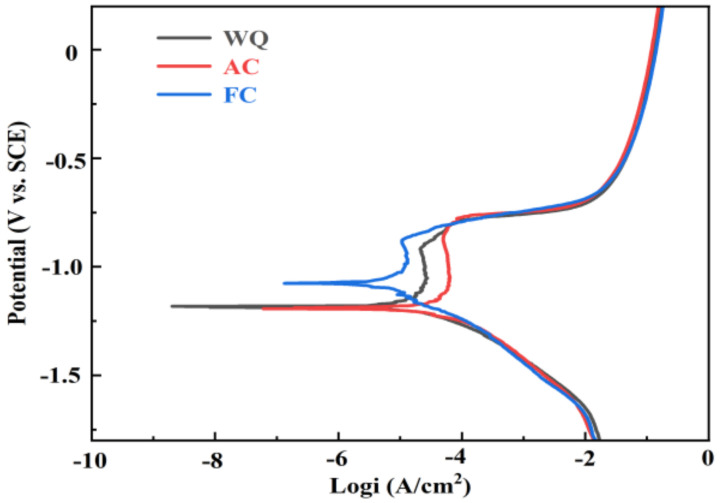
Polarization curves of Al-Zn-Mg-Cu alloys after RRA treatments with different retrogression cooling ways.

Figure 7 presents the polarization curves of samples after various RRA treatments. The linear fit and Tafel extrapolation techniques are employed based on the cathodic and anodic branches of the polarization curves [49] to calculate the corrosion potential (Ecorr) and corrosion current density (Icorr). Table 5 presents the values of Ecorr and Icorr, which are quantitatively assessed to determine the effect of retrogression cooling rate on corrosion resistance. The more positive Ecorr value suggests lower sensitivity to IGC and the lower Icorr value corresponds to a decreased corrosion rate. The findings indicate that samples treated by WQ and FC exhibit more positive Ecorr and lower Icorr compared to AC-treated samples, indicating better corrosion resistance. Meanwhile, the results of electrochemical corrosion tests were in accordance with the IGC testing results presented in Figure 6.

From the perspective of corrosion performance, FC-treated samples exhibit the best corrosion resistance. However, when comprehensively considering mechanical properties, corrosion behavior, and time cost, samples subjected to WQ treatment exhibit the best.

## 4. Discussion

### 4.1. Effect of Retrogression with Different Cooling Ways on Mechanical Properties

After the retrogression treatment with water quenching, less stable η’ and T’ dissolute, resulting in an increase in the concentration of Zn and Mg atoms within the aluminum matrix as well as an enhancement in the degree of supersaturation [50,51,52]. Subsequently, the re-aging treatment stimulates the rapid nucleation and growth of the η’ and T’ phases.

As the retrogression cooling rate decreases from WQ (Figure 2a) to AC (Figure 2b), re-precipitation of the η’ and T’ phases occurs during the relatively slow cooling process, accompanied by coarsening of residual large-sized precipitates. As a result, the size of the precipitates is larger than that of the sample after WQ treatment (Figure 2b and Table 3). Furthermore, during the air cooling process, samples did not stay for a long time between 190 ℃ and the precipitation temperature, resulting in almost no change in the proportion of η’ and T’ phases. The decrease in strength (Figure 5 and Table 4) is attributed to the coarsening of precipitates.

When the retrogression cooling rate is further reduced to FC (Figure 2c), the coarsening of precipitates intensifies, evident from a notable enlargement in precipitate size (Figure 2b and Table 3). Theoretically, this would lead to a significant reduction in strength. However, actual results demonstrate almost no change in tensile properties (Figure 5 and Table 4), which is related to the increase in the proportion of η’ phases. Generally, the nucleation and growth of precipitates occur during the aging treatment. Nucleation is influenced by preferential clustering and the growth is controlled by solute diffusion and attachment [18]. In Al-Zn-Mg-Cu alloys, the vacancies introduced during the quenching process play a crucial role in the formation of Guinier–Preston (GP) zones. GP zones enriched with solutes can be transformed into the η’ phase [53]. Mg atoms trap vacancies more easily because of their stronger vacancy bonding energy than Zn or Cu atoms [54]. At an aging temperature is 120 °C, the diffusion coefficients of Zn and Mg are 3.11 × 10^−21^ and 3.24 × 10^−21^ m^2^/s, respectively, significantly higher than that of Cu (8.40 × 10^−23^ m^2^/s) [55]. Thus, due to the size effect (with Mg atom radius being greater than Al and Zn), partial Mg-vacancies complexes preferentially cluster with Zn atoms. This process relieves the strain energy of the Al matrix, leading to the formation of Mg-Zn-vacancies complexes. Then, these Mg-Zn-vacancies (solutes-rich GP zones) gradually transform into η’ phases during the slow furnace cooling process from 190 °C and the subsequent aging process at 120 °C. This explains the increase in the phase fraction of the η’ phase. Previous research [25] has demonstrated that increasing the proportion of the η’ phase within a certain range is beneficial for improving the strength.

### 4.2. Effect of Retrogression with Different Cooling Ways on Corrosion Behavior

The micro-galvanic coupling between GBPs and the neighboring zone is recognized as the driving force of IGC [56]. This coupling typically manifests in two forms: (1) the interaction between PFZ and aluminum matrix and (2) the interplay between GBPs and PFZ. The dispersion of GBPs significantly influences the micro galvanic coupling [57]. In RRA-treated samples, the rapid nucleation process accelerates precipitate growth. This phenomenon is attributed to the high energy potential near grain boundaries during retrogression treatments [58]. Notably, GBPs do not remelt during retrogression treatments but transition towards a more stable state [50]. Consequently, GBPs aggregate, grow larger, and distribute discontinuously (Figure 4).

The corrosion resistance of Al-Zn-Mg-Cu alloys under RRA treatments varies with the retrogression cooling rate. GBPs (η’ and T’) exhibit a lower corrosion potential than the Al matrix [59], resulting in anodic dissolution of GBPs during IGC. Meanwhile, the dissolution mechanism of PFZ remains inactive. Consequently, the effect of PFZ width on the IGC of T’/η’ strengthened Al-Zn-Mg-Cu alloys is insignificant. The differences in IGC behavior depend mainly on the continuity of GBPs.

As the retrogression cooling rate was decreased from WQ to AC, GBPs underwent a certain degree of coarsening during the cooling process. At the same time, the increase in supersaturation of solute atoms induced by retrogression treatment leads to re-precipitation along grain boundaries [60]. However, the short retrogression cooling time leads to insufficient nucleation and growth of GBPs during this process, resulting in a decreased discontinuity of GBPs after re-aging treatment. Adjacent GBPs with smaller distances tend to form continuous corrosion channels, thus exacerbating corrosion.

When the retrogression cooling rate is further decreased to FC, GBPs have enough time to nucleate during the retrogression cooling process and then grow, coarsen, and distribute discontinuously during the subsequent re-aging treatment. As a result, the IGC resistance is greatly improved.

Considering the mechanical properties, IGC resistance, and time consumption, WQ is the appropriate retrogression cooling method. Notably, the performance and microstructure in this paper were obtained on laboratory-scale specimens. There may be different results for the industrial scale. In the future, it is necessary to further optimize RRA treatments for industrial profiles in order to achieve uniform performance. Moreover, it is also important to explore the coupling effects of heating rate, temperature, duration, and cooling ways during the retrogression treatment.

## 5. Conclusions

This study systematically investigates the effect of RRA treatments with different retrogression cooling ways on the microstructure evolution, mechanical properties, and IGC resistance of T’/η’ strengthened Al-Zn-Mg-Cu alloys. The main conclusions are as follows:

(1) Samples after RRA treatment with water-quenching retrogression exhibit better mechanical properties than those of air-cooled and furnace-cooled samples. The ultimate tensile strength, yield strength, and elongation of samples after RRA treatment with water-quenching retrogression are 419.2 MPa, 370.2 MPa, and 15.9, respectively. The coarsening of precipitates within grains counts for the more decreased strength of air-cooled samples than that of water-quenching samples. The similar strength of air-cooled and furnace-cooled samples is related to the size of T’/η’ precipitates within grains as well as the proportion of T’ and η’ phases;
(2) The corrosion resistance of water-quenching and furnace-cooled samples is better than that of air-cooled samples, manifested as a reduction in IGC depth and corrosion current density. The minimum IGC depth (123.7 μm) and the lowest corrosion current density (0.712 × 10^−5^ A/cm^2^) indicate the best corrosion resistance of furnace-cooled samples. The difference in IGC resistance depends on the continuity of GBPs;
(3) Taking into account the mechanical properties, corrosion resistance, and time cost, water quenching appears as a suitable retrogression way for T’/η’ strengthened Al-Zn-Mg-Cu alloys.

It should be noted that the research results presented in this paper were obtained on a laboratory scale. For industrial profiles with medium to thick dimensions, RRA treatment parameters need to be further improved to obtain uniform performance. In addition, the coupling effects of heating rate, temperature, holding time, and cooling wats during retrogression treatment remain to be explored.

## Figures and Tables

**Table 1:** Designed chemical composition range of the investigated Al-Zn-Mg-Cu alloy (wt.%).

Zn	Zn/Mg	Cu	Mn	Zr	Ti	Fe	Si	Al
5.5~6.0	2.0~2.5	≤0.5	≤0.2	0.2	0.1	≤0.5	≤0.5	Balance

**Table 2:** Details of various aging treatments.

Sample	Pre-Aging	Retrogression	Cooling Environment	Re-Aging
WQ	120 °C/24 h	190 °C/30 min	water	120 °C/24 h
AC			air	
FC			furnace	

**Table 3:** Size of precipitates within grains in the investigated Al-Zn-Mg-Cu alloy after RRA treatments with different retrogression cooling ways.

Sample	Plate-Like Precipitates			Round-Shape Precipitates
	Diameter/nm	Thickness/nm	Aspect Ratio	Diameter/nm
WQ	19.4 ± 7.4	7.9 ± 2.9	2.6 ± 0.9	7.9 ± 1.8
AC	14.3 ± 2.1	4.8 ± 0.7	3.0 ± 0.6	9.2 ± 1.4
FC	126.8 ± 19.5	32.6 ± 6.7	4.0 ± 0.9	61.9 ± 8.9

**Table 4:** Detailed tensile properties of samples after RRA treatments with different retrogression cooling ways.

Sample	UTS (MPa)	YS (MPa)	EL (%)
WQ	419.2 ± 3.0	370.2 ± 2.7	15.9 ± 1.7
AC	408.3 ± 6.5	356.2 ± 1.9	15.0 ± 1.2
FC	409.8 ± 1.7	366.5 ± 5.6	15.0 ± 1.0

**Table 5:** Electrochemical parameters of Al-Zn-Mg-Cu alloys after RRA treatments with different retrogression cooling ways.

	Ecorr (V)	Icorr (10^−5^ A/cm^2^)
WQ	−1.001 ± 0.171	0.833 ± 0.394
AC	−1.165 ± 0.038	2.363 ± 0.805
FC	−1.128 ± 0.030	0.712 ± 0.029

## Data Availability

Data will be made available on reasonable request.
